# Glucosamine supplementation in the treatment of temporomandibular joint disorders: a systematic review and meta-analysis

**DOI:** 10.3389/fdmed.2026.1868023

**Published:** 2026-07-17

**Authors:** Nancy Calzada-Gonzales, Rubén Aguirre-Ipenza, Victor Serna-Alarcón, Jordan Valeriano-Mamani, Rocio Mendoza-Torres, Miguel Hueda-Zavaleta, Alvaro Taype-Rondan, Ana Brañez-Condorena, Christoper A. Alarcon-Ruiz

**Affiliations:** 1 Escuela Profesional de Odontología, Universidad Nacional Hermilio Valdizán, Huánuco, Peru; 2 Facultad Ciencias de la Salud, Universidad Continental, Lima, Peru; 3 Escuela Profesional de Medicina Humana, Universidad Privada Antenor Orrego, Trujillo, Peru; 4 Hospital Regional José Cayetano Heredia, Seguro Social de Salud (EsSalud), Piura, Peru; 5 Escuela Profesional de Farmacia y Bioquímica, Universidad Nacional Mayor de San Marcos, Lima, Peru; 6 Escuela Profesional de Farmacia y Bioquímica, Universidad Inca Garcilaso de la Vega, Lima, Peru; 7 Dirección de Farmacovigilancia, Acceso y Uso, Dirección General de Medicamentos, Insumos y Drogas (DIGEMID), Lima, Peru; 8 Facultad de Ciencias de la Salud, Universidad Privada de Tacna, Tacna, Peru; 9 Unidad de Investigación Para la Generación y Síntesis de Evidencias en Salud, Universidad San Ignacio de Loyola, Lima, Peru; 10 EviSalud Evidencias en Salud, Lima, Peru

**Keywords:** glucosamine, meta-analysis, systematic review, temporomandibular disorders, temporomandibular osteoarthritis

## Abstract

**Background:**

Glucosamine has been proposed as a therapeutic option for temporomandibular disorders (TMD), particularly temporomandibular joint osteoarthritis (TMJ OA), but its effectiveness and safety remain uncertain.

**Objective:**

To evaluate the benefits and harms of oral glucosamine supplementation in patients with TMJ OA and other/unspecified TMD.

**Methods:**

A systematic review was conducted in PubMed/MEDLINE, CENTRAL, Scopus, and Embase through October 8, 2025. Randomized controlled trials (RCTs) evaluating oral glucosamine supplementation in patients with any type of TMD, including TMJ OA, were selected. Meta-analyses were performed using random-effects models, and the GRADE methodology was applied to assess the certainty of the evidence.

**Results:**

Seven RCTs were included. In patients with TMJ OA, compared with placebo, glucosamine may not result in a clinically important reduction in pain at ≤3 months (MD −1.58 mm; 95% CI −5.98 to 2.83; low certainty) and probably does not provide a clinically important reduction at 6 months despite statistical significance (MD −7.02 mm; 95% CI −11.95 to −2.09; moderate certainty). Glucosamine improved quality of life at 12 months (MD −4.14; 95% CI −6.41 to −1.87; high certainty). Compared with active controls, the effect on pain was uncertain, although quality of life may improve at 3 months. In patients with other/unspecified TMD, glucosamine may not meaningfully reduce pain but may increase maximum pain-free mandibular opening at 3 months. Evidence regarding adverse and serious adverse events was very uncertain across comparisons.

**Conclusion:**

Oral glucosamine does not provide a clinically important reduction in pain in TMJ OA or other/unspecified TMD but may improve quality of life in TMJ OA. Evidence for functional outcomes and adverse events remains uncertain, highlighting the need for high-quality randomized trials.

**Systematic Review Registration:**

https://www.crd.york.ac.uk/PROSPERO/view/CRD42024531131, identifier CRD42024531131

## 1 Introduction

The temporomandibular joint (TMJ) is essential for functions such as chewing, speaking, and facial expression. Temporomandibular joint disorders (TMD) affect approximately 41% of adults and 27% of children and adolescents (1). TMD can lead to various symptoms, including pain, restricted jaw movement, and clicking or popping sounds during jaw function (2). These symptoms often have significant psychological and social repercussions, limiting daily activities and social interactions, thus representing public health concern due to their substantial impact on quality of life (3, 4). Despite its prevalence and negative effects, the optimal approach for managing TMD remains unclear, warranting further investigation.

Glucosamine supplementation has been hypothesized to provide symptomatic relief for joint disorders, given its high concentration in joint tissues and its potential anti-inflammatory and cartilage-regenerative properties (5). The European Society for Clinical and Economic Aspects of Osteoporosis, Osteoarthritis, and Musculoskeletal Diseases recommends prolonged use of crystalline glucosamine sulfate as a first-line background treatment for knee osteoarthritis while discouraging the use of other glucosamine formulations (6). Since osteoarthritis can affect the temporomandibular joint (TMJ OA), a specific subtype within the broader spectrum of TMD, leading to cartilage degradation and subchondral bone remodeling, glucosamine has been proposed as a therapeutic option for this condition. However, its efficacy across the broader and often non-arthritic spectrum of TMD remains controversial, making it essential to evaluate its benefits and harms to inform clinical management strategies, considering differences between TMJ OA and other TMD.

Previous systematic reviews (7–9) provide insights into this topic; however, they exhibit notable limitations. These include the absence of clinically relevant outcomes, inadequate reporting of adverse events, and the lack of Summary of Findings (SoF) tables (10), which are key elements for synthesizing and interpreting results. Additionally, the omission of relevant studies and insufficient methodological transparency may have biased their conclusions, limiting their use during evidence-based decisions.

We conducted an exhaustive literature search, a critical evaluation of study quality, and the application of the Grading of Recommendations, Assessment, Development, and Evaluations (GRADE) approach (10, 11) to support evidence-based conclusions. Our study aims to assess the benefits (pain, mandibular opening and quality of life) and harms of glucosamine supplementation compared to placebo or active control for the treatment of TMD.

## 2 Methods

### 2.1 Protocol and registration

We conducted a systematic review and reported the results following the Preferred Reporting Items for Systematic Reviews and Meta-Analyses (PRISMA) checklist (Supplementary Table S1) to ensure transparency and rigor in our methodology (12). The study protocol was prospectively registered in the PROSPERO database under the registration number CRD42024531131.

### 2.2 Elegibility criteria

We included randomized clinical trials (RCTs) that met the following criteria: 1) Children or adults diagnosed with any type of TMD (i.e., TMJ OA or other/unspecified TMD subtypes) following any diagnostic criteria, 2) Oral supplementation with glucosamine in any formulation or as part of a co-intervention, with no restrictions on dose or treatment duration, 3) Comparisons against placebo, standard management, no treatment, or any active comparator (such as anti-inflammatories or opioids), 4) Assessment of at least one of our primary (pain, maximum pain-free or comfortable mandibular opening, and numerically measured quality of life) or secondary (maximum pain-independent opening) outcomes, 5) Outcome measurement of at least 1 month after treatment. We excluded conference abstracts.

### 2.3 Information sources and search strategy

A systematic search was performed in electronic databases, including PubMed/MEDLINE, Cochrane Central Register of Controlled Trials (CENTRAL), Scopus, and EMBASE, from inception through October 8, 2025. No restrictions were applied regarding language, publication date, study design, or publication status. Additionally, manual searches were conducted in the reference lists of the final included RCTs, and grey literature was assessed through clinical trial registries searching, such as ClinicalTrials.gov. Detailed search strategies for each database are provided in Supplementary Table S2.

### 2.4 Study selection

Duplicates were identified and removed using Rayyan software (https://www.rayyan.ai/), following standardized procedures to ensure the accuracy and integrity of the data extraction process. Six reviewers (NCG, RAI, VSA, JVM, RMT, MHZ), divided into three groups of pairs, independently conducted study selection. Each pair independently screened one-third of all full-text references. Before this, a pilot exercise was conducted to calibrate the selection criteria, achieving over 80% agreement. Study selection was performed in two phases. In the first phase, titles and abstracts of the identified studies were screened to exclude irrelevant articles. Full texts were retrieved when abstracts were unavailable or when exclusion could not be determined based on the title alone. Studies that did not meet the inclusion criteria were excluded during this phase. In the second phase, the full texts of the remaining studies were reviewed and consolidated multiple reports from the same RCT. Disagreements were resolved through discussion or, when necessary, by consulting an arbitrator (CAAR). A list of the excluded studies along with the reasons for exclusion is provided in Supplementary Table S3.

### 2.5 Data extraction and outcome measures

Two reviewers (NCG, RAI) independently extracted data for each study using Microsoft Excel, including information on study characteristics (author, year of publication, country, study design, recruitment date, funding source), population (diagnostic criteria, age range, inclusion/exclusion criteria, number and percentage of male participants), intervention (type, duration, frequency, co-interventions), and type of control (placebo, standard management, or active control), with their specific descriptions. Any discrepancies were resolved through discussion, with input from a third reviewer (CAAR) when necessary.

We systematically collected data on the study outcomes, classifying them as dichotomous or numerical variables. Dichotomous outcomes included any adverse effects and serious adverse effects (adverse events requiring hospitalization, medical intervention, or resulting in significant disability or death). Numerical outcomes comprised pain (measured on a standardized scale), maximum mandibular opening (measured in millimeters), comfortable maximum mandibular opening (achieved without pain or discomfort), and quality of life (assessed using validated questionnaires).

Initially, the assessment of crackles was considered. However, this outcome was not reported in any of the included studies.

### 2.6 Risk of bias

Pairs of reviewers (NCG, RAI, VSA, JVM, RMT, MHZ), independently and in duplicate, assess each RCT for potential biases using the Cochrane Risk of Bias (RoB) Tool (13). Each pair independently evaluated one-third of the RCTs included. Before this, a pilot exercise was conducted to calibrate the risk of bias evaluation, achieving over 80% agreement.

This evaluation covered multiple domains, including sequence generation, allocation concealment, blinding of participants, personnel, and outcome assessors, outcome data integrity (classifying ≥10% missing data classified as high risk of bias), selective outcome reporting, and other potential sources of bias, such as baseline characteristics (13). Each domain was rated as having a low, unclear, or high risk of bias based on standardized criteria from RoB 1 tool. Discrepancies were resolved through discussion, with input from a third reviewer (CAAR) when required.

### 2.7 Data synthesis and analysis

We performed meta-analyses to summarize studies that evaluated similar outcomes. The Review Manager software (version 5.4.1) was used for data collection and analysis, employing risk ratios (RR) or post-intervention mean differences (MD) with their 95% confidence intervals (95% CI). Given the heterogeneity observed among studies, random-effects models were applied.

To avoid clinical heterogeneity from pooling distinct conditions, meta-analyses were stratified and conducted separately according to the subpopulation (TMJ OA versus other/unspecified TMD). Within these populations, further subgroup analyses were conducted based on the comparator (placebo and active control) and follow-up duration (3 months or less, 6 months, and 12 months).

Heterogeneity was quantified using the I² statistic, with values below 40% considered non-relevant. It should be noted that publication bias was not assessed due to the limited number of studies included in each analysis.

### 2.8 Certainty of the evidence

Two authors (CAAR and ABC), through consensus, assessed the certainty of evidence for all reported outcomes, categorizing it as high, moderate, low, or very low following the Grading of Recommendations, Assessment, Development, and Evaluations (GRADE) approach (10, 11).

RCTs were categorized into subgroups according to diagnostic population (TMJ OA and other/unspecified TMD), comparator (placebo and active control such as ibuprofen or tramadol) and follow-up duration. The consensus process provided a comprehensive framework for evaluating imprecision, risk of bias, inconsistency, and indirectness, ensuring a thorough and contextualized assessment of these factors in line with established methodological standards (14, 15). The estimate effect of the same outcome was not downgraded for both inconsistency and imprecision. The analysis and communication of findings adhered to GRADE guidance (10, 11, 16) and was performed using the GRADEpro Guideline Development Tool (GDT) software (https://www.gradepro.org/) to create Summary of Findings (SoF).

## 3 Results

### 3.1 Study selection

**Figure 1:**
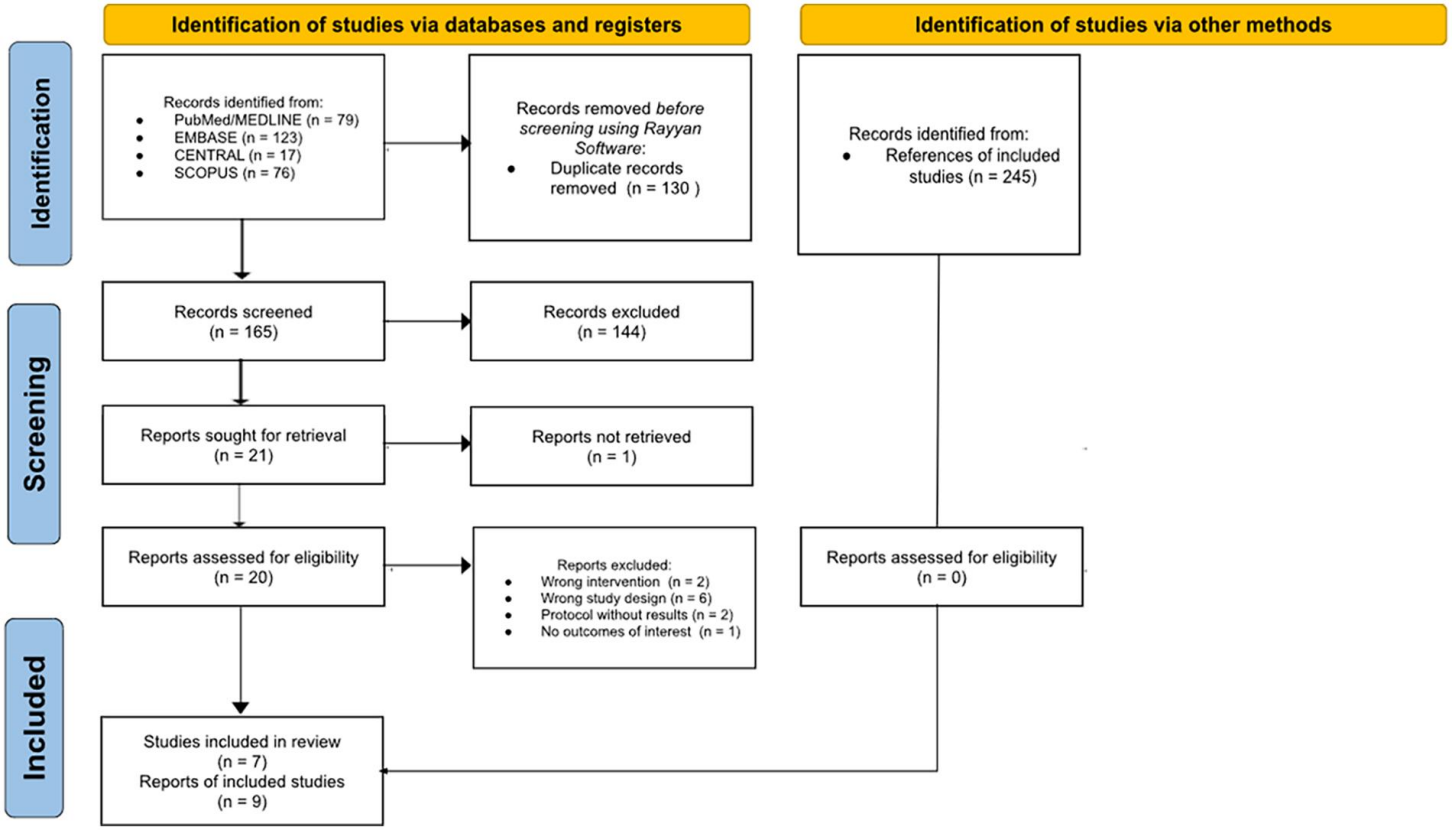
Flow diagram of studies inclusion.

We identified 295 articles in the systematic search. After eliminating duplicates, 165 records were examined by title and abstract, of which 20 were reviewed in full text. Finally, 7 RCTs were included. No additional studies were found after searching for the references for the included studies (Figure 1). Excluded studies and reasons for exclusion can be found in Supplementary Table S3.

### 3.2 Study characteristics

The included studies were published between 2001 and 2021. Two studies were conducted in Turkey (17, 18), one in Canada (19), one in the United States (20), one in Iran (21), one (with three reports) in China (22–24), and one in Sweden (25). All studies were RCTs reporting outcomes with follow-up of 3 months or less in six studies (18–21, 23, 25), 6 months in one study (23), and 12 months in two studies (17, 23).

A total of 393 participants were included, with a range of 26 to 144 per study; 87.4% were women, and the weighted average age was 39.3 years. Four studies (17, 19, 24, 25) included patients diagnosed with TMJ OA, and three studies (18, 20, 21) evaluated patients with other/unspecified TMD, four studies administered glucosamine sulfate (between 1200 and 1500 mg/day) (18, 19, 21, 25), and three administered glucosamine hydrochloride (between 1440 and 1500 mg/day) (17, 20, 24). In three studies, the control group received an active treatment (two with ibuprofen and one with tramadol) (18, 19, 21), while in other three studies, the control group received a placebo (20, 23, 25), and in one additional study the control group received a standard of care of hyaluronic acid intra/articular injection (17) (Table 1).

**Table 1:** Studies characteristics.

Author (year)	Country	Population	Sample size	Intervention	Comparator	Follow-up	Outcomes	Funding
Adults with TMJ osteoarthritis								
Johansson Chalin (25)	Sweden	Adults with TMJ osteoarthritis according to RDC/TMD Axis I, category IIIb (mean age intervention group 61.0 years, females: 86.4%).	59 (I: 3° C: 29)	Glucosamine sulfate 400 mg 3 capsules daily (Total daily dose: 1,200 mg) for 6 weeks	Placebo	6 weeks	Pain Mouth opening with pain Mouth opening without pain Any adverse event Serious adverse event	Grant support from the Gothenburg Research and Development Council and Södra Bohuslän.
Cömert (17)	Turkey	Patients with TMJ osteoarthritis according to RDC/TMD category IIIb (mean age intervention group 27.9 years, females 88.5%)	26 (I: 12 C: 14)	Glucosamine hydrochloride 750 mg, chondroitin sulfate 600 mg, and methylsulfonylmethane 350 mg, 2 doses per day (Total daily dose: 1,500 mg) for 3 months + Hyaluronic acid intra-articular injection single session	Hyaluronic acid intra-articular injection single session	12 months	Pain Maximum mouth opening Comfortable mouth opening Lateral mandibular movement Mandibular protrusion Serious adverse event	Self-funded by the authors
Thie (19)	Canada	Patients with TMJ osteoarthritis according to the American Board of Orofacial Pain 1996 including radiographic evidence of osteoarthritis and joint space narrowing (mean age 37.5 years, female: 88.9%)	39 (I: 21 C: 18)	Glucosamine sulfate 500 mg 3 times a day (Total daily dose: 1,500 mg) for 90 days	Active control (Ibuprofen 400 mg 3times a day)	3 months	Pain Mouth opening without pain. Maximum voluntary mouth opening Serious adverse event	University of Alberta Dental Fund.
Yang (22–24)	China	Adults with TMJ osteoarthritis according to RDC/TMD and the imaging results of cone-beam computed tomography (mean age intervention group 40.1 years, females: 83.3%)	144 (I: 72 C: 72)	Glucosamine hydrochloride 240 mg 2 tablets at a time, 3 times a day (Total daily dose: 1,440 mg) for 3 months. + Hyaluronate sodium 20 mg injections into the superior and inferior TMJ capsules once a week for 3 weeks	Placebo + Hyaluronate sodium 20 mg injections into the superior and inferior TMJ capsules once a week for 3 weeks	3, 6, 12 months	Pain Maximum mouth opening Lateral mandibular movement Protrusive movement and quality of life Any adverse event Serious adverse event	Funded by the Research Training Program (20091094) of Sichuan University and the National Institute of Natural Sciences. Project funding from the Foundation Innovation Group (8132100) in China.
Patients with other/unspecified TMD								
Damlar (18)	Turkey	Patients with internal derangement of TMJ (mean age 28.6 years, females: 100%)	31 (I: 16 C: 15)	Glucosamine sulfate 1,500 mg and chondroitin sulfate 1,200 mg daily for 8 weeks	Active control (tramadol HCl 50 mg twice a day)	8 weeks	Pain Maximum mouth opening Serious adverse event	Funded by the Scientific Research Support Unit of Cukurova University (Project No.: DHF2008D2).
Haghighat (21)	Iran	Patients with TMJ disorders (mean age intervention group 26.6 years, female: 76.7%) Diagnostic criteria: TMJ pain at rest, evoked pain on TMJ palpation, or TMJ clicking or noise with mandibular movement examination	60 (I: 3° C: 30)	Glucosamine sulfate 1,500 mg daily for 90 days	Active control (Ibuprofen 400 mg twice daily)	3 months	Pain Maximum comfortable mouth opening Any adverse event Serious adverse event	Self-funded by the authors
Nguyen (20)	United States	Patients with TMJ disorders (mean age intervention group 43 years, female: 88.2%) Diagnostic criteria: Pain in one or both TMJ and moderate or severe pain on lateral or dorsal palpation of the TMJ	34 (I: 14 C: 20)	Glucosamine hydrochloride 250 mg and chondroitin sulfate 200 mg, 3 tablets 2 times a day (Total daily dose: 1,500 mg) for 3 months	Placebo	3 months	Pain Any adverse event Serious adverse event	Louisiana State University Health Sciences Center School of Dentistry Research and Grants Committee

C, comparator; I, intervention; QoL, Quality of life; RDC/TMD, Research Diagnostic Criteria/Temporomandibular Disorders; TMJ, Temporomandibular joint.

### 3.3 Risk of bias

**Figure 2:**
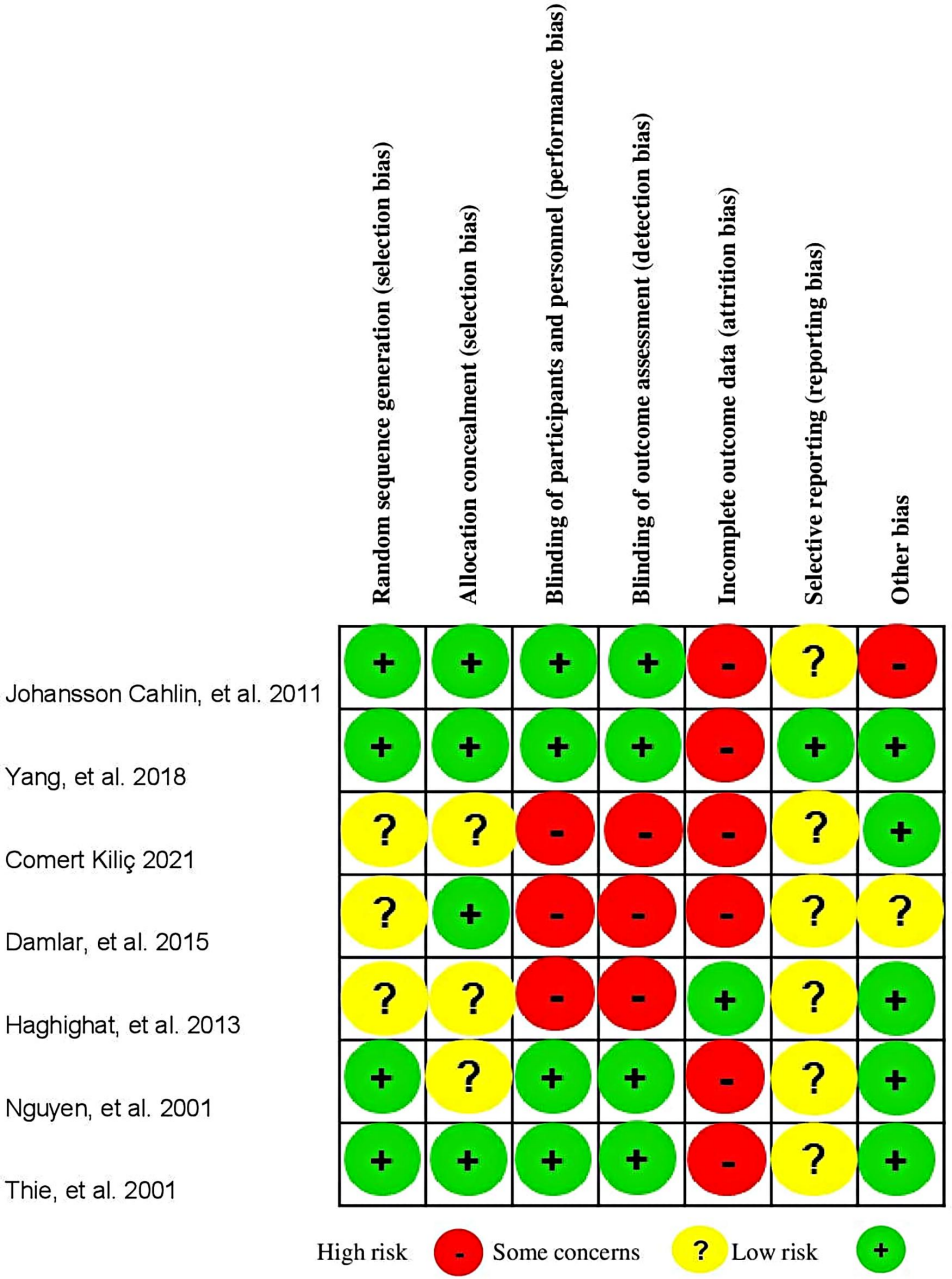
Risk of bias.

Overall, most included studies were judged to be at high risk of bias. Unclear risk of bias was identified in random sequence generation (3/7 studies), allocation concealment (3/7 studies), and selective reporting (6/7 studies). High risk of bias was observed for blinding of participants and personnel (3/7 studies), blinding of outcome assessment (3/7 studies), incomplete outcome data (6/7 studies), and other sources of bias (1/7 study) (Figure 2).

### 3.4 Results of syntheses and certainty of the evidence

**Figure 3:**
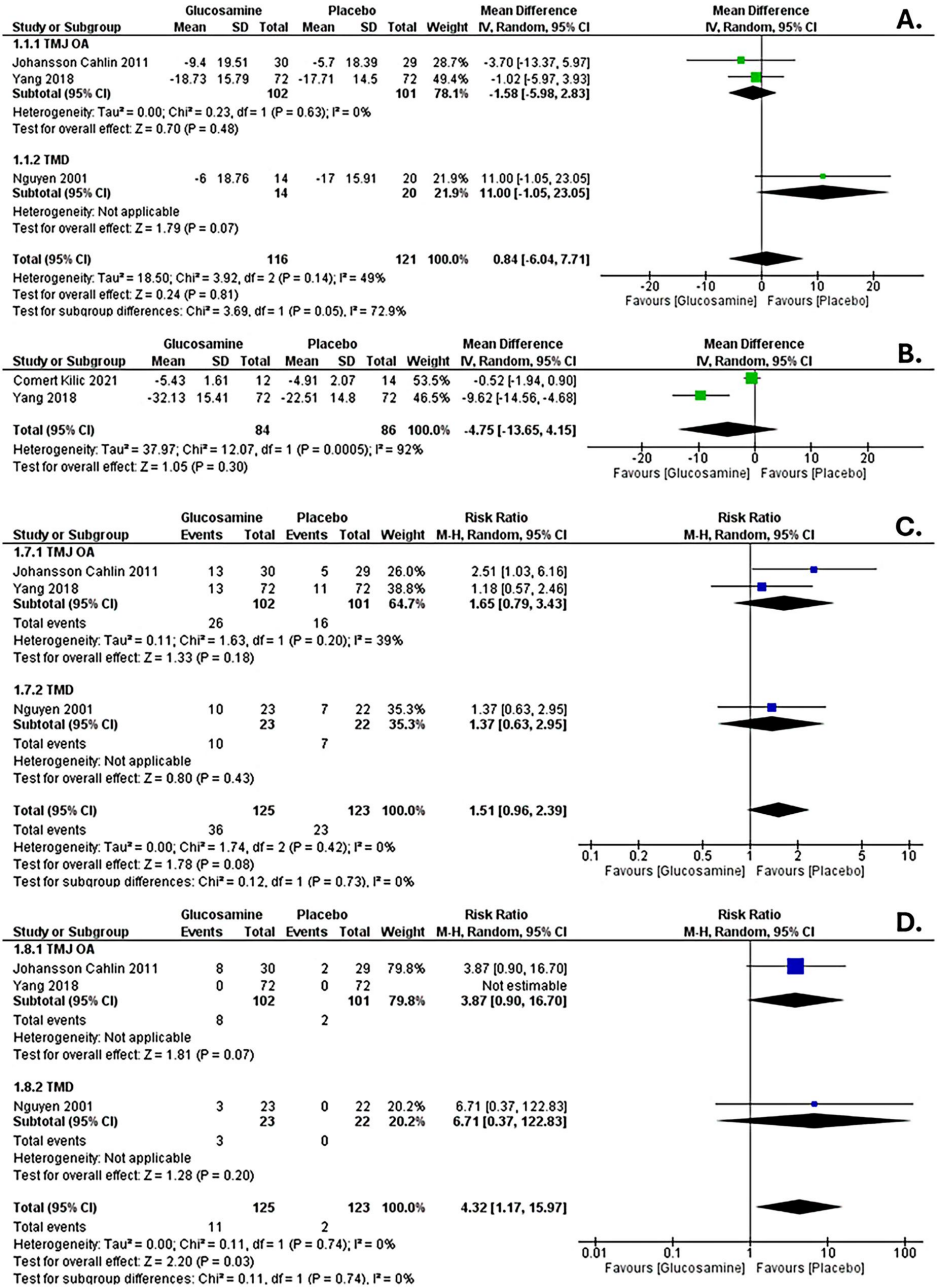
Forest plot of the comparisons between glucosamine and placebo. **(A)** Meta-analysis of pain at ≤3 months: glucosamine vs. placebo. **(B)** Meta-analysis of pain at 12 months: glucosamine vs. placebo. **(C)** Meta-analysis of any adverse event: glucosamine vs. placebo, **(D)** Meta-analysis of serious adverse events: glucosamine vs. placebo.

Meta-analyses and Summary of Findings tables summarizing the evidence on glucosamine supplementation for TMJ OA and other/unspecified TMD are presented for comparisons with placebo (Figure 3 and Table 2**)** and with active controls (Supplementary Figure S4 and Table 3).

**Table 2:** Summary of findings table for the effects of glucosamine supplementation vs. placebo in patients with TMD.

**Outcomes (follow-up)**	**Number of patients (number of studies)**	**Intervention: Glucosamine supplementation**	**Control group: Placebo**	**Relative effect (95% CI)**	**Absolute difference (95% CI)**	**Certainty**	**Interpretation**
**Patients with TMJ osteoarthritis**							
Pain (≤ 3 months)	203 (2 RCTs)	Mean: −14.07	Mean: −11.71	–	MD: −1.58 (−5.98 to +2.83)	⊕⊕◯◯	Compared to placebo, glucosamine supplementation may not cause an important effect in pain at 3 months or less of follow-up.
Visual analog scale (0 to 100 mm) with millimeters (Less is better)						Low^a^	
Pain (6 months)	144 (1 RCT)	Mean: −28.93	Mean: −21.91	–	MD: −7.02 (−11.95 to −2.09)	⊕⊕⊕◯	Compared to placebo, glucosamine supplementation probably does not importantly reduce pain at 6 months of follow-up.
Visual analog scale (0 to 100 mm) with millimeters (Less is better)						Moderate^b^	
Pain (12 months)	170 (2 RCTs)	Mean: −18.78	Mean: 13.71	–	MD: −4.75 (−13.65 to +4.15)	⊕◯◯◯	The evidence is very uncertain about the effect of glucosamine supplementation on pain at 12 months of follow-up.
Visual analog scale (0 to 100 mm) with millimeters (Less is better)						Very low^b^^,^^c^^,^^d^	
Quality of life (12 months)	144 (1 RCT)	Mean: −10.91	Mean: −6.77	-	MD: −4.14 (−6.41 to −1.87)	⊕⊕⊕⊕	Compared to placebo, glucosamine supplementation importantly improves quality of life at 12 months of follow-up.
OHIP-14 (less is better)						High	
Maximum mandibular opening without pain (Mean: 6 weeks) in milimeters	59 (1 RCT)	Mean: 2.6 mm	Mean: 0.8 mm	-	MD: +1.80 (−1.32 to +4.92)	⊕◯◯◯	The evidence is very uncertain about the effect of glucosamine supplementation on maximum painless mandibular opening at 6 weeks of follow-up.
						Very low^a^^,^^e^	
Maximum mandibular opening without pain (Mean: 12 months) in milimeters	26 (1 RCT)	Mean: 4.67 mm	Mean: 2.79 mm	-	MD: +1.88 (−3.11 to +6.87)	⊕◯◯◯	The evidence is very uncertain about the effect of glucosamine supplementation on maximum painless mandibular opening at 12 months of follow-up.
						Very low^a^^,^^e^	
Any adverse effects	203 (2 RCTs)	26/102 (25.5%)	16/101 (15.8%)	RR: 1.65 (0.79 to 3.43)	RD: 103 more for 1000 33 fewer to 385 more)	⊕◯◯◯	The evidence is very uncertain about the effect of glucosamine supplementation on adverse events.
						Very low^a^^,^^f^	
Serious adverse effects	203 (2 RCTs)	8/102 (7.8%)	2/101 (2.0%)	RR: 3.87 (0.90 to 16.70)	RD: 57 more for 1,000 (2 less to 311 more)	⊕◯◯◯	The evidence is very uncertain about the effect of glucosamine supplementation on serious adverse events.
						Very low^a^^,^^f^	
**Patients with other/unspecified TMJ disorders**							
Pain (≤ 3 months)	34 (1 RCT)	Mean: −6.00	Mean: −17.00	-	MD: +11.00 (−1.05 to +23.05)	⊕◯◯◯	The evidence is very uncertain about the effect of glucosamine supplementation on pain at 3 months of follow-up.
Visual analog scale (0 to 100 mm) with millimeters (Less is better)						Very low^a^^,^^e^	
Any adverse effects	45 (1 RCT)	10/23 (43.5%)	7/22 (31.8%)	RR: 1.37 (0.63 to 2.95)	RD: 118 more for 1,000 (118 fewer to 620 more)	⊕◯◯◯	The evidence is very uncertain about the effect of glucosamine supplementation on adverse events.
						Very low^a^^,^^g^	
Serious adverse effects	45 (1 RCT)	3/23 (13.0%)	0/22 0%	RR: 6.71 (0.37 to 122.83)	RD: 130 more for 1,000 (14 less to 1,000 more)	⊕◯◯◯	The evidence is very uncertain about the effect of glucosamine supplementation on serious adverse events.
						Very low^a^^,^^g^	

CI: confidence interval, MD, mean difference, RD, risk difference, RCT, randomized controlled trial, RR, risk ratio, TMJ, Temporomandibular joint, OHIP-14, 14-item Oral Health Impact Profile scale.

A minimal important difference (MID) was considered 8.0 mm for pain in patients with temporomandibular joint disorders (26), 1.0 points for quality of life by OHIP-14 in dental patients (27), 0.35 mm for maximum mandibular opening without pain in patients with temporomandibular joint disorders (26).

Explanations of certainty of evidence:.

a Certainty was downgraded by two levels due to the risk of bias because ≤50% of the meta-analysis' weight comes from studies with a low risk of bias.

b Certainty was downgraded by one level due to imprecision because the 95% CI of the absolute effect exceeds 1 MID.

c Certainty was downgraded by one level due to the risk of bias because 50% to 70% of the meta-analysis weight comes from studies with a low risk of bias.

d Certainty was downgraded by two levels due to inconsistency because the I² is >80%.

e Certainty was downgraded by two levels due to imprecision because the 95% CI of the absolute effect exceeds 2 MID.

f Certainty was downgraded by one level due to imprecision because the 95% CI of the relative effect exceeds 0.75 or 1.25.

g Certainty was downgraded by two levels due to imprecision because the 95% CI of the relative effect exceeds 0.75 and 1.25.

**Table 3:** Summary of findings table for the effects of glucosamine supplementation vs. active control (ibuprofen, tramadol) in patients with TMD.

**Outcomes (follow-up)**	**Number of patients (number of studies)**	**Intervention: Glucosamine supplementation**	**Control group: Active control**	**Relative effect (95% CI)**	**Absolute difference (95% CI)**	**Certainty**	**Interpretation**
**Patients with TMJ osteoarthritis**							
Pain (≤ 3 months)	39 (1 RCT)	Dif of mean: −10.5	Dif of mean: −5.93	–	MD: −4.57 (−9.91 to +0.77)	⊕◯◯◯	The evidence is very uncertain about the effect of glucosamine supplementation on pain at 3 months of follow-up.
Visual analog scale (0 to 100 mm) with millimeters (Less is better)						Very low^a^^,^^b^	
Quality of life (3 months)	39 (1 RCT)	Dif of mean: −15.07	Dif of mean: −8.33	–	MD: −6.74 (−14.70 to +1.22)	⊕⊕◯◯	Compared to active control, glucosamine supplementation may importantly improve quality of life at 3 months of follow-up.
						Low^b^	
Brief Pain Inventory Questionnaire (Less is better)							
Maximum mandibular opening without pain (Mean: 3 months) in millimeters	39 (1 RCT)	Dif of mean: +10.14 mm	Dif of mean: +8.39 mm	–	MD +1.75 (−4.10 to +7.60)	⊕◯◯◯	The evidence is very uncertain about the effect of glucosamine supplementation on maximum painless mandibular opening at 3 months of follow-up.
						Very low^a^^,^^c^	
Any adverse effects	45 (1 RCT)	2/23 (8.7%)	3/22 (13.6%)	RR: 0.64 (0.12 to 3.46)	RD: 49 fewer per 1,000 (120 fewer to 335 more)	⊕◯◯◯	The evidence is very uncertain about the effect of glucosamine supplementation on adverse events.
						Very low^a^^,^^d^	
Serious adverse effects	45 (1 RCT)	2/23 (8.7%)	3/22 (13.6%)	RR: 0.64 (0.12 to 3.46)	RD: 49 fewer per 1,000 (120 fewer to 335 more)	⊕◯◯◯	The evidence is very uncertain about the effect of glucosamine supplementation on serious adverse events.
						Very low^a^^,^^d^	
**Patients with other/unspecified TMJ disorders**							
Pain (≤ 3 months)	91 (2 RCTs)	Dif of mean: −3.9	Dif of mean: −3.11	–	MD: −0.90 (−1.88 to +0.07)	⊕⊕◯◯ Low^a^	Compared to active control, glucosamine supplementation may not have an important impact in pain at 3 months or less of follow-up.
Visual analog scale (0 to 100 mm) with millimeters (Less is better)							
Maximum mandibular opening without pain (Mean: 3 months) in millimeters	60 (1 RCT)	Dif of mean: +6.76 mm	Dif of mean: +3.53 mm	-	MD +3.23 (+0.82 to +5.64)	⊕⊕◯◯ Low^a^	Compared to active control, glucosamine supplementation may increase the maximum mandibular opening without pain at 3 months of follow-up.
Any adverse effects	66 (1 RCT)	5/33 (15.2%)	16/33 (48.5%)	RR: 0.31 (0.13 to 0.75)	RD: 335 fewer per 1,000 (422 fewer to 121 fewer)	⊕◯◯◯	The evidence is very uncertain about the effect of glucosamine supplementation on adverse events.
						Very low^a^^,^^e^	
Serious adverse effects	66 (1 RCT)	0/33 (0%)	0/33 (0%)	Not estimated	Not estimated	⊕⊕◯◯ Low^a^	Not estimated

CI, confidence interval, MD, mean difference, RCT, randomized controlled trial, RD, risk difference, RR, risk ratio, TMJ, Temporomandibular joint.

A minimal important difference (MID) was considered 8.0 mm for pain in patients with temporomandibular joint disorders (26) 4.0 points for quality of life by Brief Pain Inventory-Facial in patients with trigeminal neuralgia (28), 0.35 mm for maximum mandibular opening without pain in patients with temporomandibular joint disorders (26).

**Explanations of certainty of evidence:.**

a Certainty was downgraded by two levels due to the risk of bias, as ≤50% of the meta-analysis weight consists of studies with a low risk of bias.

b Certainty was downgraded by two levels due to imprecision because the 95% CI of the absolute effect exceeds 2 MID.

c Certainty was downgraded by one level due to imprecision, as the 95% CI of the absolute effect exceeds 1 MID.

d Certainty was downgraded by two levels due to imprecision because the 95% CI of the relative effect exceeds 0.75 and 1.25.

e Certainty was downgraded by one level due to imprecision, because the 95% CI of the relative effect exceeds 0.75 or 1.25.

#### 3.4.1 Outcome: pain

Pain was assessed using the visual analog scale (VAS, 0–100 mm), with lower scores indicating less pain (29). Although some variability in VAS administration was reported across studies, all included RCTs used a standardized 0–100 mm scale, ensuring comparability of effect estimates.

In patients with TMJ OA, compared with placebo, glucosamine supplementation at ≤3 months follow-up may not result in an important reduction in pain (2 RCTs; MD: −1.58 mm; 95% CI: −5.98 to 2.83; low certainty of evidence) **(**Figure 3A**)**, and at 6 months of follow-up probably does not produce an important improvement in pain compared with placebo (1 RCT; MD: −7.02 mm; 95% CI: −11.95 to −2.09; moderate certainty of evidence), as although the effect is statistically significant, it does not exceed the minimal important difference of 8 mm. At 12 months of follow-up, the evidence is very uncertain (2 RCTs; MD: −4.75 mm; 95% CI: −13.65 to +4.15; very low certainty of the evidence) **(**Figure 3B) (Table 2).

In patients with other/unspecified TMD, compared with placebo at ≤ 3 months of follow-up, the impact of glucosamine supplementation in pain is very uncertain (1 RCT; MD: +11.0 mm; 95% CI: −1.05 to +23.05; very low certainty of the evidence) **(**Figure 3A).

Regarding the comparison with active control, at ≤3 months of follow-up, the effect of glucosamine supplementation on pain in patients with TMJ OA is very uncertain (1 RCT; MD: −4.57 mm; 95% CI: −9.91 to +0.77; very low certainty of the evidence), while in patients with other/unspecified TMD may not have an important impact (2 RCTs; MD: −0.90 mm; 95% CI: −1.88 to +0.07; low certainty of the evidence) (Supplementary Figure S4A).

#### 3.4.2 Outcome: quality of life

In patients with TMJ OA, glucosamine supplementation importantly improves quality of life, measured using the Oral Health Impact Profile-14 (OHIP-14) questionnaire, at 12 months compared to placebo (1 RCT; MD: −4.14; 95% CI: −6.41 to −1.87; high certainty of the evidence). When compared to active control, glucosamine supplementation may importantly improve quality of life, as assessed with the Brief Pain Inventory questionnaire at 3 months (1 RCT; MD: −6.74; 95% CI: −14.70 to +1.22; low certainty of the evidence). There was no evidence for this outcome on patients with other/unspecified TMD.

#### 3.4.3 Outcome: maximum mandibular opening without pain

In patients with TMJ OA, the evidence is very uncertain about the effect of glucosamine supplementation on maximum pain-free mandibular opening compared to placebo, both at 6 weeks follow-up (1 RCT; MD: +1.8 mm; 95% CI: −1.32 to +4.92; very low certainty of the evidence) and at 12 months follow-up (1 RCT; MD: +1.88 mm; 95% CI: −3.11 to +6.87; very low certainty of the evidence). When comparing glucosamine supplementation to active control, the evidence regarding its effect on maximum pain-free mandibular opening at 3 months of follow-up is very uncertain (1 RCT; MD: +1.75 mm; 95% CI: −4.10 to +7.60; very low certainty of the evidence) (Supplementary Figure S4B).

In patients with other/unspecified TMD, the glucosamine supplementation may increase the maximum pain-free mandibular opening at 3 months of follow-up, compared to active control (1 RCT; MD: +3.23 mm; 95% CI: +0.82 to +5.64) (Supplementary Figure S4B).

#### 3.4.4 Outcome: adverse events

In patients with TMJ OA, compared to placebo, the evidence is very uncertain about the effect of glucosamine supplementation on any adverse events [2 RCTs; risk difference (RD): +103 events per 1,000 patients, 95% CI: −33 to +385; very low certainty of the evidence] (Figure 3C) and serious adverse effects (2 RCTs; RD: +57 events per 1,000 patients, 95% CI: −2 to +311; very low certainty of the evidence) (Figure 3D). While, in patients with other/unspecified TMD, compared to placebo, the evidence is also very uncertain about the effect of glucosamine supplementation on any adverse events (1 RCT; RD: +118 events per 1,000 patients, 95% CI: −118 to +620; very low certainty of the evidence) (Figure 3C) and serious adverse effects (1 RCT; RD: +130 events per 1,000 patients, 95% CI: −14 to +1,000; very low certainty of the evidence) (Figure 3D).

In patients with TMJ OA, when comparing glucosamine supplementation with active control, the evidence is very uncertain on adverse events (1 RCT; RD: −49 events per 1,000 patients, 95% CI: −120 to +335; very low certainty of the evidence) (Supplementary Figure S4C) and serious adverse effects (1 RCT; RD: −49 events per 1,000 patients, 95% CI: −120 to +335; low certainty of the evidence) (Supplementary Figure S4D). While in patients with other/unspecified TMD, the evidence is very uncertain on adverse events (1 RCT; RD: −335 events per 1,000 patients, 95% CI: −422 to −121; very low certainty of the evidence) (Supplementary Figure S4C) and not reported any serious adverse effects (1 RCT; low certainty of the evidence) (Supplementary Figure S4D).

## 4 Discussion

### 4.1 Summary of main results

Overall, the evidence suggests that glucosamine does not provide a clinically important reduction in pain compared with placebo or active controls in TMJ OA and in other/unspecified TMD, with effects generally small and of low to very low certainty, particularly in the short and medium term. In TMJ OA, compared with placebo, the effect in pain at 6 months did not reach the minimal important difference, and evidence at 12 months was very uncertain. For other/unspecified TMD, the effect of glucosamine supplementation on pain was highly uncertain. In contrast, glucosamine showed a clinically important improvement in quality of life in patients with TMJ OA at 12 months compared with placebo, while evidence vs. active controls was limited and of low certainty. Evidence regarding maximum pain-free mandibular opening was very uncertain in TMJ OA, with a possible small improvement observed only in patients with other/unspecified TMD compared with active controls at 3 months. Finally, the evidence on adverse and serious adverse events was very uncertain across all comparisons, precluding firm conclusions about the safety profile of glucosamine in these populations.

### 4.2 Certainty of the evidence

The overall certainty of the evidence was very low for most outcomes, largely driven by methodological limitations of the included trials and imprecision of the effect estimates. A high risk of bias was common, particularly related to inadequate blinding, incomplete outcome data, and unclear allocation concealment, all of which may have led to overestimation or underestimation of treatment effects. Specifically, risk of bias was downgraded by one level for most pain outcomes (≤3 months and 6 months in TMJ OA, and pain outcomes in other/unspecified TMD), by two levels for pain at 12 months due to the predominance of high risk of bias studies and imprecision, and no downgrade was applied for quality of life at 12 months because the estimate was derived from a single study judged to have low risk of bias across most domains. In addition, most studies enrolled relatively small numbers of participants, resulting in wide confidence intervals that often encompassed both clinically important benefits and no effect. This imprecision limits confidence in the observed estimates and hinders definitive conclusions regarding the effectiveness and safety of glucosamine supplementation in TMJ OA and other/unspecified TMD.

Furthermore, the limited number of trials per comparison and outcome restricted the ability to explore heterogeneity or conduct robust subgroup analyses, further contributing to uncertainty. Diagnostic heterogeneity may also have contributed to indirectness, as studies used different criteria to define TMJ OA and other/unspecified TMD conditions. This variability may reduce confidence in the applicability of the findings across different clinical settings and patient populations.

Collectively, as previous reviews have suggested (8, 30), these limitations highlight the need for larger, well-designed randomized controlled trials with adequate blinding, standardized outcome measures, and sufficient follow-up to provide more precise and reliable estimates of the effects of glucosamine in these populations.

### 4.3 Pain and quality of life

Glucosamine is classified as a symptomatic slow-acting drug for osteoarthritis (SYSADOA) and several mechanisms have been proposed, including modulation of cartilage metabolism, attenuation of pro-inflammatory mediators, and restoration of joint homeostasis (31, 32). However, these mechanisms are largely derived from preclinical studies and evidence from osteoarthritis in other joints, and their clinical relevance in TMJ OA remains uncertain (6, 30, 33, 34). The current clinical evidence included in this review does not demonstrate a clinical important reduction in pain and therefore does not provide strong support for a clinically relevant effect of these proposed biological mechanisms in TMJ OA.

However, the available evidence suggests that oral glucosamine does not result in a clinically important reduction in pain; however, it may lead to improvements in quality of life in patients with TMJ OA and other/unspecified TMD.

The discrepancy between pain and quality-of-life outcomes may be explained by the multidimensional nature of quality of life, which captures aspects beyond pain intensity, including function, emotional well-being, and social participation. Even small changes in pain or functional symptoms that do not reach the minimal important difference may still translate into perceived improvements in daily functioning. Additionally, improvements in quality of life may reflect dimensions beyond pain intensity, including perceived functional status, emotional well-being, and social participation. However, the mechanisms underlying this finding remain uncertain and should be interpreted cautiously given the low certainty of the evidence (35). Methodological limitations, such as small sample sizes and risk of bias, may also have reduced the ability to detect differences in pain outcomes while allowing changes in broader patient-reported measures.

### 4.4 Mandibular opening

Patients with TMD commonly experience pain-related functional limitations, particularly during mandibular opening, which adversely affect daily activities and quality of life (36). The findings regarding mandibular opening are inconclusive, as potential benefits were observed only in patients with other/unspecified TMD and only when glucosamine was compared with active controls. Accordingly, previous systematic reviews suggest that glucosamine sulfate provides only modest effects on pain and maximal mandibular opening, with results that are not consistently statistically significant and considerable methodological heterogeneity (including formulation, dosage, treatment duration, and co-interventions) (8, 37).

This may be explained by differences in pathophysiology between TMJ OA and other/unspecified TMD, as functional limitation in non-osteoarthritic TMD may be more influenced by muscle dysfunction than by structural joint damage (6, 38). These differences further support the decision to analyze TMJ OA and other/unspecified TMD populations separately throughout this review, as combining these populations could obscure clinically relevant differences in treatment effects. Also, the fact that this effect was observed only in comparison with active controls may reflect differences in comparator efficacy rather than a true treatment effect. However, since these results are based on a small number of trials with very low certainty of evidence, warranting cautious interpretation.

### 4.5 Adverse events

The evidence regarding adverse events is highly uncertain, primarily driven by the limited number of trials, small sample sizes, and inconsistent or incomplete reporting of safety outcomes across studies.

Several trials were not designed or powered to detect adverse or rare serious events, resulting in wide confidence intervals and imprecise effect estimates. In addition, variability in the definitions and ascertainment of adverse events, as well as short follow-up periods in most studies, further limits the ability to draw reliable conclusions regarding the safety profile of glucosamine. Consequently, the available evidence does not allow for definitive conclusions about the presence or absence of an increased risk of adverse or serious adverse events, underscoring the need for larger, well-designed trials with standardized and systematic safety reporting.

### 4.6 Overall completeness and applicability of evidence

The included studies focused on adult populations diagnosed with TMD, with a predominant representation of women (18, 20, 23, 25). No studies were found in pediatric populations or in older adults with comorbidities, which may limit the generalizability of the findings to a more diverse population and indicate a gap in evidence for these groups. Studies included patients with other/unspecified TMD (18, 20, 21) and TMJ OA (17, 19, 23, 25). However, not all studies applied uniform diagnostic criteria, which may compromise comparability and affect the ability to draw consistent conclusions across studies.

The diagnostic approaches varied substantially across studies and included RDC/TMD-based classifications, American Board of Orofacial Pain criteria with radiographic confirmation, cone-beam computed tomography (CBCT)-based diagnostic criteria, and studies relying primarily on clinical manifestations. Such variability may introduce indirectness because participants classified under the same broad diagnostic category may not represent equivalent clinical populations. Consequently, the observed treatment effects may not be fully comparable across studies and should be interpreted with caution.

Furthermore, TMJ OA and other/unspecified TMD subtypes differ in their underlying pathophysiology, clinical manifestations, and potentially their response to treatment. Because glucosamine is primarily proposed as a structure-modifying and symptom-modifying intervention for osteoarthritic conditions, its effects may differ between degenerative joint disease and non-osteoarthritic TMD conditions. For this reason, we analyzed TMJ OA and other/unspecified TMD populations separately and avoided pooling these populations within the same meta-analyses.

Glucosamine supplementation was evaluated in two main formulations: glucosamine sulfate (18, 19, 21, 25) and glucosamine hydrochloride (17, 20, 23). Comparators included placebo (20, 23, 25) and active controls such as ibuprofen (19, 21), tramadol (18), and hyaluronic acid (17), contributing to variability in results. However, the lack of studies directly comparing different glucosamine formulations limits conclusions regarding their relative efficacy. Additionally, studies assessing long-term effects, safety outcomes, and functional improvements are scarce. Another important limitation relates to the use of co-interventions. Several studies evaluated glucosamine supplementation in combination with chondroitin sulfate, methylsulfonylmethane (MSM), or intra-articular hyaluronic acid injections rather than standalone intervention. As a result, the observed treatment effects may reflect the combined effect of multiple therapies, limiting the ability to isolate the specific contribution of glucosamine.

Long-term studies on glucosamine use in TMD treatment are scarce. Most studies reported follow-up durations of 6 to 12 weeks (19, 20, 21, 25). Only one study (17, 23) provided data with a 12-month follow-up, which limits the ability to assess the sustained effects of supplementation conclusively. Additionally, there is a lack of studies evaluating patient-reported outcomes, such as quality of life and long-term pain relief, which are critical for assessing the real-world impact of glucosamine in TMD.

Additionally, variations in glucosamine formulations and control groups introduced inconsistencies that may have influenced treatment effects and complicated direct comparisons (39). While some RCTs suggest a potential benefit of glucosamine, these methodological limitations prevent definitive conclusions. Addressing these issues requires well-powered RCTs with standardized interventions and rigorous bias control to more reliably assess its benefits and harms in TMD.

### 4.7 Agreements and disagreements with other studies or reviews

Our systematic review identifies key differences compared to previous studies. Derwich (32) concluded that it is insufficient to confirm the clinical effectiveness of glucosamine in TMJ OA, highlighting that benefits were primarily associated with treatment duration, with significant improvements in mandibular opening and pain reduction reported only after three months. Melo (8) found that glucosamine was comparable to ibuprofen in reducing pain over 12 weeks; however, its effectiveness remained uncertain when administered for six weeks, showing no significant differences from placebo. Similarly, a Cochrane review 2012 (40) reported no significant differences in pain or mandibular opening between ibuprofen and glucosamine sulfate, although a meta-analysis could not be conducted due to the limited number of included studies. In contrast, our systematic review applied a broader analysis with rigorous methodological criteria, including the GRADE system, to assess the certainty of the evidence.

Machado (41) suggested that glucosamine and chondroitin sulfate may be effective and safe for TMJ OA; however, this conclusion was based on only two RCTs, substantially limiting its robustness and generalizability. In contrast, our systematic review included seven RCTs with varying formulations and treatment durations, providing a more comprehensive assessment. Unlike previous systematic reviews, our analysis differentiated effects according to comparator type (placebo vs. active treatment) and follow-up duration, allowing for a more detailed evaluation of glucosamine supplementation in the clinical management of TMD.

### 4.8 Clinical importance

From a clinical perspective, the available evidence suggests that oral glucosamine is unlikely to provide a clinically meaningful reduction in pain for patients with TMJ OA or other temporomandibular disorders and therefore should not be recommended as an analgesic treatment for these conditions. However, the potential improvement observed in quality of life indicates that some individuals may perceive benefits in overall well-being or daily functioning, even in the absence of substantial pain relief.

The interpretation of clinical relevance was based on an anchor-based minimal important difference (MID) of approximately 8 mm on the visual analog scale, derived from patients with chronic TMD. Although some clinicians may consider more conservative thresholds (e.g., 10–20 mm) depending on baseline symptom severity and clinical context, our interpretation of clinical importance was robust to these alternative thresholds. Specifically, the only statistically significant effect on pain observed in this review (MD −7.02 mm at 6 months vs. placebo in TMJ OA) did not reach the selected MID and would likewise fail to meet higher thresholds of clinical importance.

Given the low certainty of evidence for most outcomes and the uncertainty regarding adverse events, clinicians should carefully weigh patient preferences, costs, and alternative evidence-based interventions when considering glucosamine supplementation. Overall, current findings support a cautious, individualized approach and highlight the need for shared decision-making until more robust evidence becomes available.

### 4.9 Limitations and strengths

We identified the following limitations: Despite efforts to control variability through subgroup analyses, heterogeneity was observed among the included studies, potentially due to differences in glucosamine dosages (ranging from 1200 to 1500 mg), study populations (Asia, Europe, and America), and quality-of-life assessment criteria (Brief Pain Inventory questionnaire, OHIP-14). Some studies had methodological limitations, such as small sample sizes (17–20). Clinical heterogeneity was also present due to differences in diagnostic categories, although this was partially addressed by conducting separate analyses for TMJ OA and other/unspecified TMD populations. An additional limitation is that several trials evaluated combination therapies rather than glucosamine alone. The use of co-interventions such as chondroitin sulfate, MSM, and hyaluronic acid injections introduces potential confounding and complicates attribution of treatment effects specifically to glucosamine. Furthermore, the small number of studies within each diagnostic criteria or co-intervention category prevent an additional subgroup analysis according to further population or intervention composition.

However, this study also has several strengths. We applied the GRADE methodology to assess the certainty of the evidence and present the summary of findings. A comprehensive search was conducted across multiple databases without language or date restrictions, minimizing selection bias and enhancing the generalizability of the findings. Unlike previous systematic reviews that focus solely on glucosamine benefits, our systematic review evaluates both benefits and harms, providing a more comprehensive assessment for clinical decision-making.

## 5 Conclusions

The current evidence does not support a clinically important effect of oral glucosamine on pain reduction in patients with TMJ OA or other/unspecified TMD. While improvements in quality of life were observed, particularly in TMJ OA, these findings should be interpreted with caution given the low certainty of evidence for most outcomes and the methodological limitations of the included trials. Evidence regarding functional outcomes, such as pain-free mandibular opening, and adverse events remains uncertain.

## Data Availability

The original contributions presented in the study are included in the article/Supplementary Material, further inquiries can be directed to the corresponding author.

## Supplementary material

Supplementary Figure S4 Forest plot of the comparisons between glucosamine and active control, for TMJ OA and TMD. (S4A) Meta-analysis of pain at ≤3 months: glucosamine vs. active control. (S4B) Meta-analysis of maximum mandibular opening without pain at ≤3 months: glucosamine vs. active control. (S4C) Meta-analysis of any adverse events: glucosamine vs. active control (S4D) Meta-analysis of serious adverse events: glucosamine vs. active control.
