# Supplementary material for: Glucosamine supplementation in the treatment of temporomandibular joint disorders: a systematic review and meta-analysis
Source: Front Dent Med. 2026 Jul 17;7:1868023. doi: 10.3389/fdmed.2026.1868023 (PMC13423845; doi:10.3389/fdmed.2026.1868023)

**Supplementary Figure S4. Forest plot of the comparisons between glucosamine and active control, for TMJ OA and TMD.**

(S4A) Meta-analysis of pain at ≤ 3 months: glucosamine vs. active control.


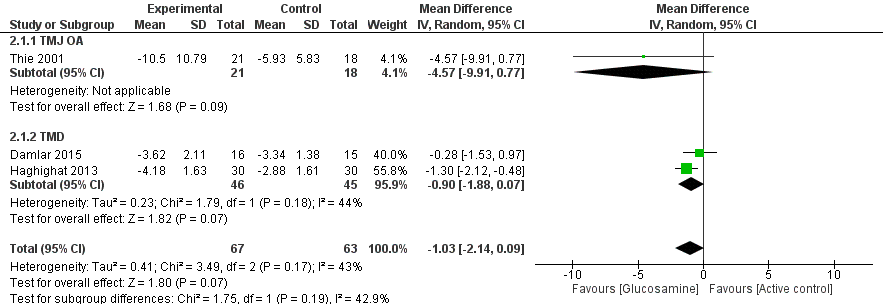


(S4B) Meta-analysis of maximum mandibular opening without pain at ≤ 3 months: glucosamine vs. active control.


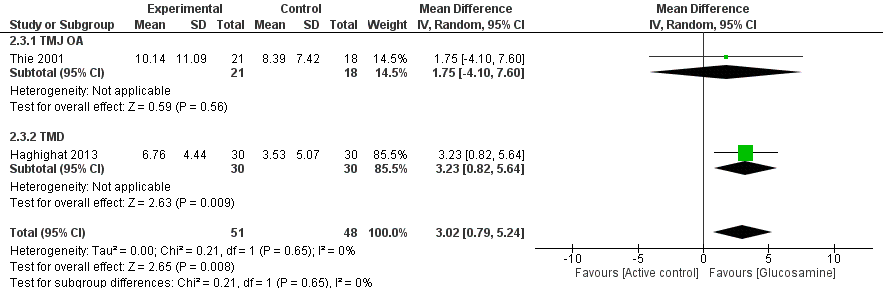


(S4C) Meta-analysis of any adverse events: glucosamine vs. active control


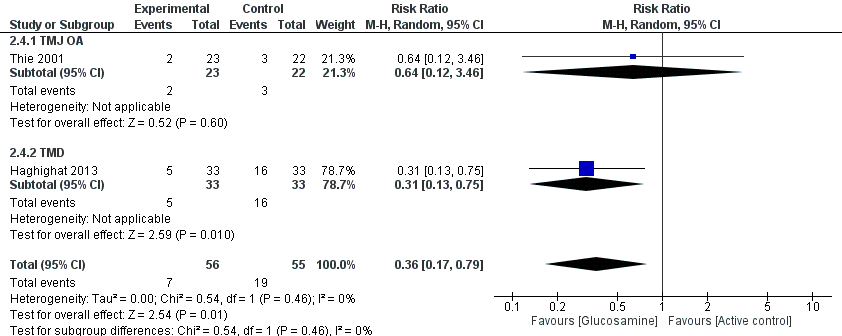


(S4D) Meta-analysis of serious adverse events: glucosamine vs. active control


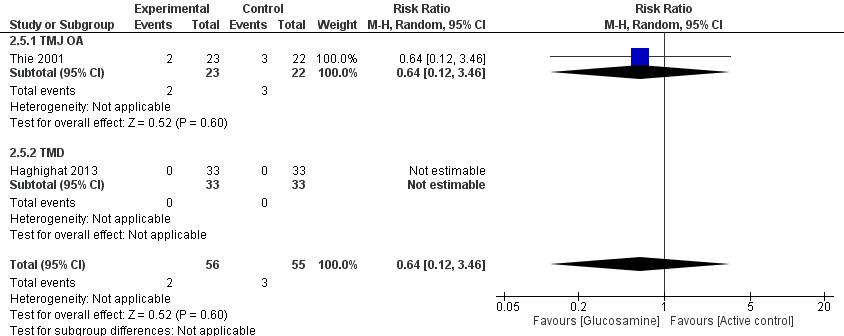

Supplement: Supplementary Figure S4 — Forest plot of the comparisons between glucosamine and active control, for TMJ OA and TMD. (S4A) Meta-analysis of pain at ≤3 months: glucosamine vs. active control. (S4B) Meta-analysis of maximum mandibular opening without pain at ≤3 months: glucosamine vs. active control. (S4C) Meta-analysis of any adverse events: glucosamine vs. active control (S4D) Meta-analysis of serious adverse events: glucosamine vs. active control. [file Table4.docx]
