# Supplementary material for: Glucosamine supplementation in the treatment of temporomandibular joint disorders: a systematic review and meta-analysis
Source: Front Dent Med. 2026 Jul 17;7:1868023. doi: 10.3389/fdmed.2026.1868023 (PMC13423845; doi:10.3389/fdmed.2026.1868023)
Supplement: Supplementary file 3 [file Table2.docx]

## **Supplementary Table 2. Detailed search strategy for four databases**

Date of search: October 8th, 2025

**Search in PubMed MEDLINE**

| **Database** | **Search date:** October 8th, 2025 | | **Results** |
| --- | --- | --- | --- |
| **Search strategy** | #1 | (Temporomandibular* OR "temporo mandibular" OR TMJ OR craniomandibular OR craneomandibular OR mandibulotemporal OR mandibular OR "Costen Syndrome" OR "Costen's Syndrome" OR Jaw) AND (glucosamine* OR acetylglucosamine* OR "2-Amino-2-Deoxyglucose" OR Hespercorbin OR Dona OR "Dona S" OR Xicil OR alateris OR aminodeoxyglucose OR arafisio OR chitosamine OR "glucose amine" OR flexea OR flexove OR glucomed OR glucosamil OR "nsc 758") | 79 |

**Search in Cochrane Library**

| **Database** | **Search date:** October 8th, 2025 | | **Results** |
| --- | --- | --- | --- |
| **Search strategy** | #1 | Mesh descriptor: [Temporomandibular Joint] explode al trees | 367 |
|  | #2 | Mesh descriptor: [Temporomandibular Joint Disorders] explode al trees | 1173 |
|  | #3 | Mesh descriptor: [Jaw Diseases] explode al trees | 3374 |
|  | #4 | Temporomandibular* OR "temporo mandibular" OR TMJ OR craniomandibular OR craneomandibular OR mandibulotemporal OR mandibular OR "Costen Syndrome" OR "Costen's Syndrome" OR Jaw | 14997 |
|  | #5 | Mesh descriptor: [Glucosamine] explode al trees | 314 |
|  | #6 | glucosamine* OR acetylglucosamine* OR "2-Amino-2-Deoxyglucose" OR Hespercorbin OR Dona OR "Dona S" OR Xicil OR alateris OR aminodeoxyglucose OR arafisio OR chitosamine OR "glucose amine" OR flexea OR flexove OR glucomed OR glucosamil OR "nsc 758" | 1096 |
|  | #8 | (#1 OR #2 OR #3 OR #4) AND (#5 AND #6) | 17 |
|  | #1 | Mesh descriptor: [Temporomandibular Joint] explode al trees | 367 |

**Search in Scopus**

| **Database** | **Search date:** October 8th, 2025 | | **Results** |
| --- | --- | --- | --- |
| **Search strategy** | #1 | 'temporomandibular joint'/exp OR 'jaw disease'/exp OR Temporomandibular* OR "temporo mandibular" OR TMJ OR craniomandibular OR craneomandibular OR mandibulotemporal OR mandibular OR "Costen Syndrome" OR "Costen's Syndrome" OR Jaw AND  TITLE-ABS-KEY(temporomandibular* OR "temporo mandibular" OR tmj OR craniomandibular OR craneomandibular OR mandibulotemporal OR mandibular OR "Costen Syndrome" OR "Costen's Syndrome" OR jaw ) AND TITLE-ABS-KEY( glucosamine* OR acetylglucosamine* OR "2-Amino-2-Deoxyglucose" OR hespercorbin OR dona OR "Dona S" OR xicil OR alateris OR aminodeoxyglucose OR arafisio OR chitosamine OR "glucose amine" OR flexea OR flexove OR glucomed OR glucosamil OR "nsc 758" ) | 123 |

**Search in EMBASE**

| **Database** | **Search date:** October 8th, 2025 | | **Results** |
| --- | --- | --- | --- |
| **Search strategy** | #1 | 'temporomandibular joint'/exp OR 'jaw disease'/exp OR Temporomandibular* OR "temporo mandibular" OR TMJ OR craniomandibular OR craneomandibular OR mandibulotemporal OR mandibular OR "Costen Syndrome" OR "Costen's Syndrome" OR Jaw AND  'glucosamine'/exp OR glucosamine* OR acetylglucosamine* OR "2-Amino-2-Deoxyglucose" OR Hespercorbin OR Dona OR "Dona S" OR Xicil OR alateris OR aminodeoxyglucose OR arafisio OR chitosamine OR "glucose amine" OR flexea OR flexove OR glucomed OR glucosamil OR "nsc 758" | 76 |
