# Supplementary material for: Glucosamine supplementation in the treatment of temporomandibular joint disorders: a systematic review and meta-analysis
Source: Front Dent Med. 2026 Jul 17;7:1868023. doi: 10.3389/fdmed.2026.1868023 (PMC13423845; doi:10.3389/fdmed.2026.1868023)
Supplement: Supplementary file 4 [file Table3.docx]

## **Supplementary Table 3. Excluded studies.**

| **Author and year** | **Title of the study** | **Exclusion reason** |
| --- | --- | --- |
| Sharma, 2011 | Glucosamine and Chondroitin Sulfate versus Arthrocentesis in internal derangement of TM joint: A 5 years experience. | Wrong study design |
| Ahmedov, 2006 | The role of chondroprotectors in combined treatment of temporomandibular joint disorders | No access to fulltext |
| Alhayek, 2023 | Evaluation of Local Application of Glucosamine Sulfate and Chondroitin Sulfate on Temporomandibular Joint Response and Alleviation of Pain and Tension During the Functional Treatment of Skeletal Class II Patients: A Randomized Control Clinical Trial | Wrong intervention |
| Brignardello-Petersen, 2018 | There seems to be similar improvement in pain and mouth opening limitation when comparing glucosamine supplements with a placebo or ibuprofen in patients with temporomandibular joint osteoarthritis | Wrong study design |
| Ctri. | Evaluation of glucosamines in temporomandibular disorders | Protocol without results (from registries) |
| Chi, Ctr Trc. | Glucosamine Hydrochloride combined with Hyaluronate Sodium for Temporomandibular Joint Disorders: a Randomized Controlled Trial | Protocol without results (from registries) |
| Drks. | Effect of Combined Glucosamine Sulfate and Chondroitin Sulfate Application on Functional Treatment Efficacy of Skeletal Class II Malocclusion | Wrong intervention |
| Ganti, 2018 | Evaluation of Effect of Glucosamine-Chondroitin Sulfate,  Tramadol, and Sodium Hyaluronic Acid on Expression of Cytokine  Levels in Internal Derangement of Temporomandibular Joint | Wrong study design |
| Reginster, 2012 | Role of glucosamine in the treatment for osteoarthritis | Wrong study design |
| Surya Sudhakar, 2018 | Long-Term management of temporomandibular joint degenerative changes and osteoarthritis: an attempt | No outcomes of interest |
| Shankland, 1998 | The effects of glucosamine and chondroitin sulfate on osteoarthritis of the TMJ: a preliminary report of 50 patients | Wrong study design |
| Wood, 2008 | Temporomandibular disorders | Wrong study design |
